# Soft Tooling-Friendly Inductive Mold Heating—A Novel Concept

**DOI:** 10.3390/mi12040454

**Published:** 2021-04-17

**Authors:** Tobias Vieten, Davide Zanin, Andrea Knöller, Thomas Litwin, Wolfgang Eberhardt, André Zimmermann

**Affiliations:** 1 University of Stuttgart, Faculty for Engineering Design, Production Engineering and Automotive Engineering, Institute for Micro Integration (IFM), Allmandring 9b, 70569 Stuttgart, Germany; d.zanin@gmx.de (D.Z.); zimmermann@ifm.uni-stuttgart.de (A.Z.); 2 Hahn-Schickard, Allmandring 9b, 70569 Stuttgart, Germany; Andrea.Knoeller@Hahn-Schickard.de (A.K.); Wolfgang.Eberhardt@Hahn-Schickard.de (W.E.); 3 University of Stuttgart, Faculty for Engineering Design, Production Engineering and Automotive Engineering, Institute of Design and Production in Precision Engineering, Pfaffenwaldring 9, 70569 Stuttgart, Germany; thomas.litwin@ikff.uni-stuttgart.de

**Keywords:** soft tooling, inductive heating, variothermal injection molding, molded interconnect device

## Abstract

In order to economize injection molded prototypes, additive manufacturing of, e.g., curable plastics based tools, can be employed, which is known as soft tooling. However, one disadvantage of such tools is that the variothermal process, which is needed to produce polymeric parts with small features, can lead to a shorter lifespan of the tooling due to its thermally impaired material properties. Here, a novel concept is proposed, which allows to locally heat the mold cavity via induction to circumvent the thermal impairment of the tooling material. The developed fabrication process consists of additive manufacturing of the tooling, PVD coating the mold cavity with an adhesion promoting layer and a seed layer, electroplating of a ferromagnetic metal layer, and finally patterning the metal layer via laser ablation to enhance the quality and efficiency of the energy transfer as well as the longevity by geometric measures. This process chain is investigated on 2D test specimens to find suitable fabrication parameters, backed by adhesion tests as well as environmental and induction tests. The results of these investigations serve as proof of concept and form the base for the investigation of such induction layers in actual soft tooling cavities.

## 1. Introduction

Molded interconnect devices (MIDs) are three-dimensional circuit carriers, which can, among other things, outperform conventional printed circuit boards (PCBs) due to an increased geometrical as well as circuit design freedom. The MID substrates are typically fabricated by injection molding (IM), allowing complex geometries for MIDs with a high integration density. The subsequent maskless production steps, such as laser direct structuring (LDS) as well as the wet chemical metallization, enable creating individualized conductive patterns based on laser activatable additives in the thermoplastic materials [1], which can be further functionalized in a final step by different assembly techniques.

IM is a manufacturing method for polymeric parts and generally profitable only in mass-production, as it typically requires a hardened steel mold, which is time consuming and expensive to fabricate. One approach to speed up the tool fabrication and lower the costs is to use milled aluminum inserts instead. While this approach is applicable to produce small numbers of conventional plastic parts, it is not applicable for the production of MIDs. During IM, abrasive wear occurs in the aluminum mold, contaminating the surface of MID substrates. This aluminum contamination then acts as a catalyst in the electroless plating process, resulting in an uncontrolled overplating [2]. Another approach to fabricate metal tooling is powder bed fusion (PBF) additive manufacturing (AM). Typically, PBF inserts have to be milled to improve the cavity surface roughness, but a recent study shows, that it could be possible to use them without additional post processing [3]. By manufacturing mold inserts from polymers (e.g., thermoset resins), metal contamination and, thus, rejects due to overplating can be avoided. Such inserts are called soft tooling and can be produced in various ways, one of which is additive manufacturing [4]. There is a wide variety of additive manufacturing technologies which are categorized by ISO/ASTM 52900 into seven groups, but the most common technologies used for the production of soft tooling are vat photopolymerization (VPP), material jetting (MJT), and material extrusion (MEX). VPP tooling was used in studies to, e.g., evaluate the producibility of micro features [5,6,7], investigate its failure mechanisms [8,9] and compare the performance of VPP tooling with MJT tooling [10,11]. The MJT tooling performance was also compared to conventional metal tooling [12,13], PBF AM tooling [12] and resin casting tooling [14]. MEX tooling, or more specific fused filament fabrication (FFF) tooling, was studied, e.g., for use in metal injection molding [15] and also used in a study describing the development of a process-specific FFF soft tooling material [16]. The soft tooling inserts are comparable to milled aluminum tools in terms of their cost and fabrication-time. However, soft tooling has the advantage of enabling a higher design freedom, e.g., to add complex internal cooling channels. On the other hand, fewer parts can be produced with soft tooling compared to aluminum tooling and the comparably low thermal conductivity of polymers makes internal tempering features, such as cooling channels or heating cartridges, less effective. The latter is especially critical when producing parts that have a high surface to volume ratio (typically parts with high aspect ratios or small parts, [17,18,19]). Exposing the melt to an insufficiently heated cavity leads to partial solidification and increased flow resistance for following material up to the point, at which the melt flow path is plugged and the cavity cannot be completely filled, resulting in incomplete parts [20]. To counteract this effect, the cavity must have an adequate temperature control. During injection of the melt, the cavity has to be hot enough to avoid early solidification, but once the mold is filled, the temperature needs to be lowered to enable a controlled solidification. Such a process is called variothermal [20]. Furthermore, the influence temperature and flow of the melt have on the crystallization of, e.g., polypropylene was previously shown by experiment and simulation [21]. The simplest strategy to apply a temperature control is to heat/cool the entire insert. However, due to the poor thermal conductivity of polymer inserts, the external heating required to sufficiently heat the cavity walls would make the insert more susceptible to damage due to its thermally impaired material properties and reduce the total number of parts that can be produced with one set of inserts [12]. More promising, the novel heating concept proposed in this paper makes use of the high electromagnetic permeability of polymers. By lining the cavity walls with a ferromagnetic metal layer (e.g., nickel) and exposing it to an alternating electromagnetic field, the cavity walls can be heated inductively, while minimizing the thermal load on the rest of the insert. For realizing this concept, two major challenges need to be addressed. Such a hybrid material, composed of a polymer substrate and metal layer, will encounter different kinds of stress during the IM cycles. First, there is thermomechanical stress. The metal typically has a significantly lower coefficient of thermal expansion (CTE) than the polymer, which causes the two linked materials to expand differently during heating and, vice versa, to contract differently during cooling. This CTE mismatch, thus, induces mechanical tensile stress in the thin metal layer, which could cause cracks or delamination. A second source of mechanical failure of the metal layer is a phenomenon called “eggshell” or “thin ice” effect, which describes cracking and chipping of a thin hard coating on a soft substrate under the application of local forces [22].

In order to cope with these challenges, the presented concept comprises vat photopolymerization AM using a resin appropriate for soft tooling, physical vapor deposition (PVD) to deposit an adhesion promoting layer, as well as a seed layer for the subsequent electroplating with nickel and a final laser ablation step to divide the nickel layer into mechanically and electrically decoupled tiles. This process chain aims to enable:

- A sufficient adhesion between polymer substrate and metal layer, which prevents delamination.
- A reduction of thermomechanical stress locally (for each tile) and globally (for the nickel layer overall).
- The elimination of the eggshell effect without permitting melt to access the laser ablated grooves during the IM process.
- That each square forms an individual electric eddy current, when being exposed to an alternating magnetic field, to achieve a homogenous inductive heating.

These raised points shall be verified by adhesion tests as well as environmental and induction tests on the developed plated and patterned 2D test specimens.

## 2. Materials and Methods

Two types of substrate material were used in this study. First and more important, Prodways Materials PLASTCure Rigid 10500, a UV-curable resin filled with spherical silica particles (diameter from 120 nm to 5 µm) [23]. The resin was chosen due to its thermal stability and comparably low thermal expansion. The CTE of about 30 ppm/K below the glass transition temperature (T_G_) at 90 °C and 90 ppm/K over T_G_ was determined by thermomechanical analysis (Netzsch TMA 202, Selb, Germany). Vat photopolymerization AM was performed using an industrial digital light processing (DLP) machine (Prodways ProMaker L5000). 2D test specimens (37 mm × 54 mm × 1.2 mm) were produced, which allowed evaluating the process parameters. Prior to further processing, these test specimens were cleaned, using an ultrasonic bath with isopropyl alcohol, and post-cured in a UV-chamber.

The second material used was the thermoplastic liquid crystal polymer (LCP) Vectra E840i LDS [24] for evaluating the laser ablation process, as this process is fully independent from the underlying substrate material. Due to its darker color, the LCP material allowed for easier process inspection via optical microscopy compared to PLASTCure Rigid 10500. Plates with dimensions comparable to the above mentioned 2D test specimens were obtained via IM. The optical microscope used at various stages of this study was a Nikon SMZ1270.

### 2.1. Physical Vapor Deposition (PVD)

The adhesion between nickel and polymer depends on the material combination itself as well as on the condition of the substrate surface. Therefore, two different adhesion promoting layers and an oxygen plasma pretreatment (OPP) were tested. The test specimens were coated, using a magnetron-sputtering unit (Creamet 750 CL2), with either 20 nm titanium, 20 nm chromium, or without an adhesion promoting layer. All three alternatives were produced with and without an OPP. A second PVD step was carried out to deposit a 200 nm nickel-vanadium (NiV) layer, which is essential to initiate the following electroplating process. All specimens were then electroplated with nickel as described in the following section. The relevant process parameters are listed in Table 1.

The quality of the adhesion was evaluated by cross-cuts (acc. to DIN EN ISO 2409), followed by tape-tests. Ten fields consisting of 6 × 6 cuts with 1 mm spacing were cut with the help of a template and a utility knife. Tape-tests were carried out with Tesa tesakrepp 4308 Blue Krepp (tape A) and 3M Scotch Filament Klebeband 890MSR (tape B), which have an adhesion strength of 4 N/cm and 8.1 N/cm, respectively, on steel. The number of tiles remaining on the specimens was counted after cross-cutting, after testing with tape A and after testing with tape B. This was done manually aided by the magnification of an optical microscope.

### 2.2. Electroplating Process

After PVD coating, the test specimens were electroplated on standard electroplating equipment made by Walter Lemmen GmbH, Kreuzwertheim, Germany. Nickel was chosen for its ferromagnetic properties, as it is, thus, heated by the alternating magnetic field not only via the induction of eddy currents but also via hysteretic and excess losses [25,26]. Nickel was plated according to the process parameters listed in Table 1 to obtain layer thicknesses of 15 µm, 50 µm, and 100 µm. The actual thickness of the electroplated nickel layers was verified by micrometer gauge measurements and computer tomography (X-Tek HMXST160).

### 2.3. Laser Ablation

Test specimens were produced as described in Section 2.2 with nickel layer thicknesses of 15 µm, 50 µm, and 100 µm. A picosecond-pulsed Nd:YVO_4_ laser with a wavelength of 532 µm and a spot size of 23 µm was used for the laser ablation process. The scan speed was set to 1.4 m/s and the pulse frequency to 200 kHz, which results in a lengthwise pitch of 7 µm. The transversal pitch was adjusted to match the lengthwise pitch. In order to determine the best laser ablation parameters, 2.5 mm long rectangular cuts were made with a width of 25 µm, 50 µm, 100 µm, and 200 µm, respectively. The laser power was varied between 2, 4, 6, and 8 W, and the number of passes was increased in variable steps until the desired penetration to the polymer substrate could be observed under an optical microscope. The results were also examined by scanning electron microscopy (Jeol JSM-6490lv) and the cut edge (frayed or smooth) as well as separation quality and defects were assessed to sort out parameter combinations. From the remaining parameter combinations we selected the best based on economic aspects.

### 2.4. Reliability Tests

The PLASTCure Rigid 10500 test specimens were produced with the optimum parameters of all previous investigations: Substrate fabrication via DLP AM, oxygen-plasma pretreated and PVD-coated with 20 nm Ti as well as 200 nm NiV. Nickel was electroplated in two thicknesses, 15 µm and 50 µm. A layout with 4 fields consisting of 25 square tiles with 3 mm edge length was laser ablated, using the parameters described in Section 2.3 and a cut width of 50 µm. The laser power was set to 6 W and the number of passes was chosen to completely separate the nickel tiles from each other. The procedure of ablating the described layout is from here on referred to as “structuring”.

A first set of tests was conducted in a CTS CV-70/350-10S thermal cycle oven. The test specimens were exposed to 80 cycles of temperatures changes between 40 and 80 °C with heating/cooling rates of 8 K/min and 10 min of dwell time. The temperature interval was chosen to emulate the temperature changes the bulk material of soft tooling inserts was exposed to in previous tests. The tape-tests were conducted with tape A followed by tape B, before the test as well as after the 80 cycles. The results were examined via optical microscopy.

A second set of tests was conducted in a CTS TSS-70/130 thermal shock oven to emulate the injection molding cycles. The two chambers were set to 80 °C and 180 °C, thus crossing T_G_ every cycle. The dwell time in each chamber was set to 10 min. This dwell time allowed the specimens to fully adopt the ambient temperature in each cycle, which increases the chance of adhesion failure. The test specimens were exposed to 160 cycles. Before the test as well as after 10, 20, 40, 80, and 160 cycles tape-tests were conducted with tape A followed by tape B (see Section 2.3). The results were likewise examined via optical microscopy.

### 2.5. Inductive Heating

The test specimens were produced as described in Section 2.4 with three different nickel layer thicknesses (15 µm, 50 µm, and 100 µm), with and without laser structuring. In order to improve the quality of the thermographic examination, all specimens were painted with matte black lacquer.

For the evaluation of the heating effect, the nickel layer was exposed to an alternating electromagnetic field which induced eddy currents and was thus heated according to Joule’s first law, which describes the relationship between the power dissipation *P*, the electrical current *I* and the electrical resistance *R* of the conductor as

(1) $$P\sim I^{2}\cdot R.$$

**Figure 1:**
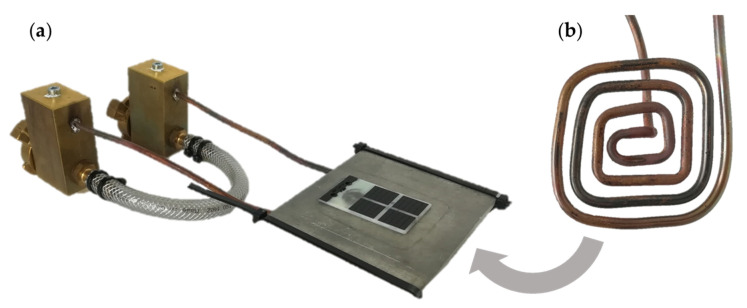
Testing equipment for inductive heating: (**a**) Test setup consisting of a test specimen on an insulation board with the inductor placed below; hookup system for electricity and coolant (water); (**b**) Pancake inductor fabricated from 8 mm copper tubing.

The high frequency generator (Trumpf Hüttinger TruHeat HF 5010) ran in an automatic mode with 35 A nominal current, 1200 V nominal voltage and variable power up to 11.2 kW. The inductor was fabricated from 8 mm copper tubing and designed as a pancake coil with parallel straight vertical and horizontal segments. Coil and test setup are shown in Figure 1. The thermal response of the nickel layer was observed with a thermographic camera, calibrated to measure temperatures from −40 °C up to 150 °C (Flir A655SC).

## 3. Results

### 3.1. Physical Vapor Deposition of Seed Layer

**Figure 2:**
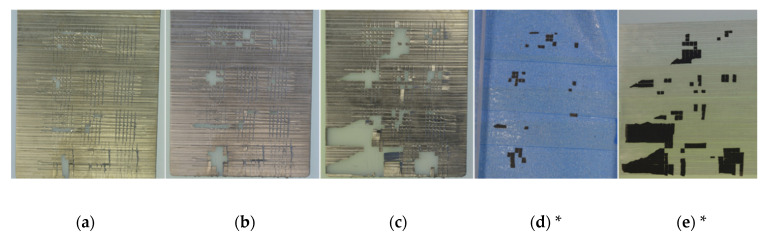
Pictures of a test specimen (Cr/NiV physical vapor deposition layer with previous oxygen plasma pretreatment) after different adhesion tests stages, tile size is 1 mm × 1 mm: (**a**) after cross-cut adhesion test; (**b**) after tape-test with tape A; (**c**) after tape-test with tape B; (**d**) corresponding tape A with adhering nickel tiles; (**e**) corresponding tape B with adhering nickel tiles. (*: images are flipped horizontally to highlight the complementary character of specimen and corresponding piece of tape).

Avoiding delamination can be achieved by tailoring the interface between polymer substrate and nickel layer. The test specimens (PLASTCure Rigid 10500) were processed and tested as described in Section 2.1 and Section 2.2. Due to the abundance of similar looking specimens, Figure 2 shows the testing progress by the example of an oxygen plasma pretreated (OPP) and Cr/NiV-coated specimen as well as the respective tape sections with the adhering nickel tiles.

**Figure 3:**
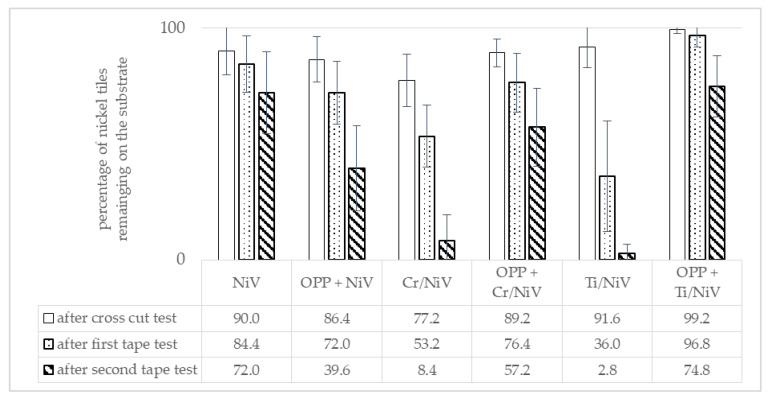
Results of the cross-cut and tape-tests for investigated combinations of adhesion promoting layer and oxygen plasma pretreatment (OPP). The columns represent the amount of nickel tiles remaining on the substrate after each test. Error bars represent the 95% confidence interval.

The results of the adhesion tests are summarized in Figure 3. It can be seen that the process resulting in the strongest adhesion of nickel on the polymer substrate is an OPP, followed by PVD coating of Ti/NiV. Specimens processed in this way had an average of about 75% of nickel tiles still adhering on the substrate after all three tests. After the second tape-test, the untreated specimens (NiV only) showed comparably good results with an average of 72% nickel tiles remaining on the substrate. However, a comparison after the first tape test shows an advantageous behavior of specimens with OPP and a Ti adhesion promoting layer. Because of the overall best performance, OPP and PVD coating of a 20 nm layer of Ti as adhesion promoting layer was set as the default procedure for the following investigations.

### 3.2. Laser Ablation of Electroplated Nickel Layer

**Figure 4:**
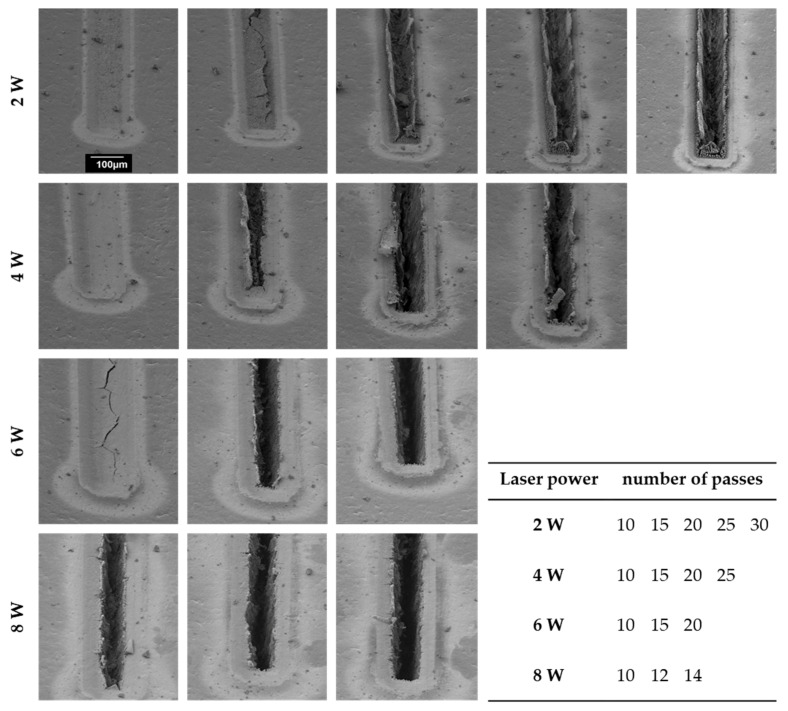
Scanning electron microscope images of laser-ablated nickel layers with varying laser power and corresponding numbers of passes. The images show a test specimen with a 15 µm thick electroplated nickel layer and a cut width of 100 µm.

Aside from a good cutting quality, i.e., well-defined geometry and complete mechanical and electrical decoupling of the tiles, a high ablation rate and minimum ablation area are desirable to achieve lower processing times and to avoid that molten polymer can access the structure during IM. Cut widths of 25, 50, 100, and 200 µm were tested, but because of the laser spot size of 23 µm, no sufficient separation of the nickel layer could be achieved for the smallest cut width of 25 µm. The minimum cut width was, therefore, defined as 50 µm. The results of the ablation process on a test specimen (Vectra E840i LDS) with an electroplated nickel layer of 15 µm are shown in Figure 4, exemplarily for a cut width of 100 µm. The top row displays results obtained with a laser power of 2 W and increasing passes from left to right, according to the table in the bottom right corner. It can be seen that the separation of the nickel layer began with a crack formation in the center of the cutting layout. The remaining material on both sides of the crack then bended upwards and remained along the flanks of the cut even after the following passes. A similar behavior could be also detected when using 4 W, which is displayed in the second row. In contrast, using 6 W and 8 W, ablation was successful, resulting in defined edges after 14 and 20 passes, respectively. For the two remaining cut widths (50 and 200 µm), the results were qualitatively similar. The best geometric quality was always observed when using 6 W laser power.

The effect of upward-bending material was only observed on specimens electroplated with 15 µm nickel. For 50 µm and 100 µm thick electroplated nickel layers, the edge quality was good throughout all tested laser parameters. However, in these cases, the cut resembled the shape of a “V”. Generating mostly perpendicular flanks could only be achieved by increasing the number of passes, which was accompanied by partial ablation of the polymer substrate. For 8 W laser power, the partial ablation of the polymer substrate led to the formation of bubbles, inducing delamination of the nickel layer from the substrate. Table 2 contains the numbers of passes required to cut through the electroplated nickel layer depending on laser power, thickness of the layer, and cut width. The width of the cut had no significant effect, except for the lowest layer thickness of 15 µm, at which a broader cut led to a reduced ablation.

**Figure 5:**
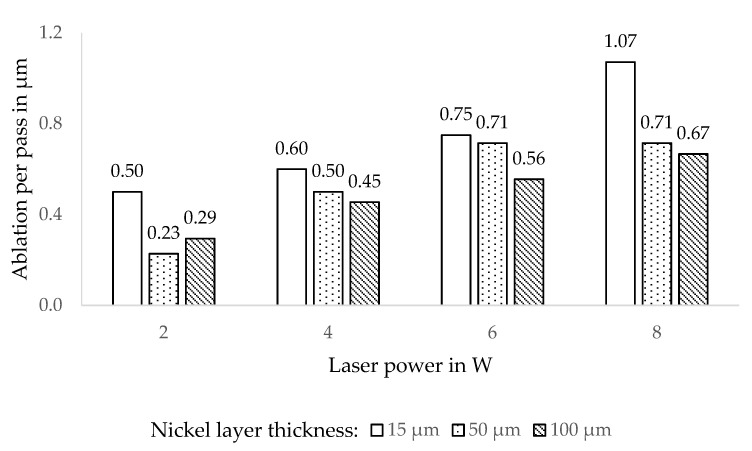
Nickel thickness ablated in one pass depending on laser power and nickel layer thickness. The cut width for these data points is 100 µm.

Defining the average ablation per passage, which is the nickel layer thickness divided by the number of passes needed for a completed cut, allowed directly comparing samples and laser-parameters, thus evaluating the effectivity of the laser ablation. This parameter is plotted in Figure 5 in dependence of laser power and nickel layer thickness for an exemplary cut width of 100 µm. The ablation per passage varies with laser power as is expected, but also with nickel layer thickness. This discrepancy originates on the one hand from the aforementioned formation of a ‘V’ shaped cut, which required more passes in the case of samples with a higher layer thickness. On the other hand, in a thicker nickel layer, the heat energy spreads faster into the adjacent material, thus lowering the temperature in the ablation zone, which reduces the amount of ablated material for the following laser pulse.

Altogether, the best among the tested laser power settings was determined to be 6 W at which defined cuts were generated with a reasonable number of passes along with the avoidance of delamination between polymer substrate and nickel layer.

### 3.3. Reliability Tests

**Figure 6:**
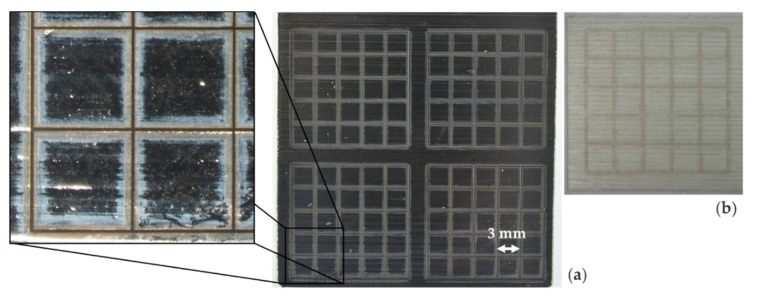
Example of a test specimen (physical vapor desposition coated, electroplated and structured; cut width 50 µm) for temperature change testing: (**a**) Photo of the specimen and detail view via microscopy; (**b**) Tape with adhering laser ablation residue after the tape-test.

After identifying suitable sets of the tested PVD and laser ablation process parameters, the tape-tests on PLASTCure Rigid 10500 specimen did not show any detached tiles on tapes A and B. Only some residue from the ablation process was visible on the tape as shown in Figure 6a,b. The same residue appeared after all tape-tests performed during temperature change testing. The tape-test performed after 80 cycles showed equal results and the microscopy inspection showed no visible damage of the nickel structure. The strong adhesion could also be preserved after 10, 20, 40, 80, and 160 cycles of the thermal shock test. Likewise, the microscopy inspection showed no visible damage of the nickel structure.

As there was no damage noticeable in any of the temperature change or thermal shock tests, there was also no difference in the results in relation to electroplated layer thickness (15 µm and 50 µm). Therefore, both layer thicknesses qualified for the following inductive heating tests.

### 3.4. Inductive Heating

**Figure 7:**
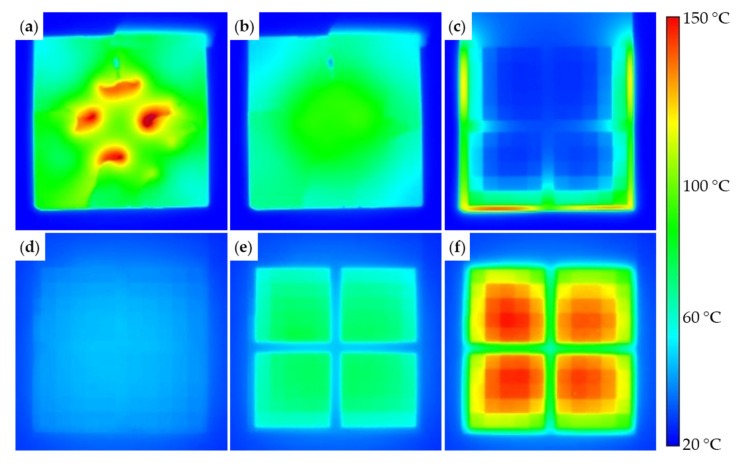
Thermal images of the inductive heating tests, tile size is 3 mm × 3 mm: (**a**) unstructured specimen in the inhomogeneous field; (**b**) the same specimen two seconds after deactivating the generator; (**c**) structured specimen at 50 W generator power; (**d**–**f**) structured specimen with unstructured nickel removed at 11.2 kW generator power after 0 s, 1 s, and 9 s, respectively.

When applying an alternating electromagnetic field, the generally thin nickel layers only permit a reduced internal heat transfer, thus building an inhomogeneous heat map. Even after shutting down the inductor, the heat does not spread evenly over the nickel-plated area but is instead emitted to the environment by convection and radiation. This can be seen in Figure 7, which presents the thermal images of a test specimen (PLASTCure Rigid 10500) at the moment the inductor was turned off (a) and two seconds after (b). The four thermal hot spots correspond to the four parallel winding sectors of the inductor, which result in a concentrated electromagnetic field. The issue of uneven heating is eliminated by structuring the nickel layer. Testing a structured specimen (as presented in Figure 6) in the electromagnetic field showed a concentration of the heating effect surrounding the structured area. As an example, Figure 7c features a specimen (50 µm nickel layer, structured) after being exposed to the alternating field for 15 s at 50 W generator power. The heating takes place in an area on the outermost edge of the (unstructured) nickel layer while the structured areas in the center are still at room temperature. This behavior can be explained with Ampère’s circuital law, which describes the relationship between the line integral of a magnetic field and the electric current that flows through the area enclosed by the line:

(2) $$\oint_{s}^{}\vec{B}\cdot ds=I\cdot {µ}_{0},$$

with *s* representing an arbitrary closed loop, the magnetic flux density *B*, the electric current *I*, and the vacuum permeability µ_0_. Assuming *B* is approximately constant over the considered area, the induced current in the larger connected area surrounding the structured fields is greater than in every separate tile, leading to a faster heating. By removing the unstructured areas of the nickel layer, only equally large areas remain, resulting in a more homogeneous heating, which is illustrated in Figure 7d–f over the course of about 10 s. In both experiments, the nickel tiles reached temperatures of about 150 °C. It is noteworthy, that the power had to be raised to 11.2 kW (maximum possible setting of the inductor) in the case of the structured specimen, although both had the same nickel layer thickness of 50 µm. With decreasing layer thickness, a reduction in the transfer of power could be identified, which is expected according to eddy current theory [27]. To reach a similar temperature within a 15 µm and a 100 µm nickel layer the power of the generator had to be increased 32-fold (11.2 kW) while also doubling the exposure duration.

The maximum temperature of 150 °C shown in Figure 7 originates from the calibration range of the camera (−40 to 150 °C) but is not the maximum achievable temperature of the heating setup. Although we were not able to measure temperature values over 150 °C, we observed the base material of specimens heated to the point of smoking, which, based on a previously performed thermogravimetric analysis (Netzsch STA 409C, Selb, Germany), should only happen well above 200 °C.

To summarize the inductive heating experiments, structuring the nickel layer is necessary for a uniform heating and the unstructured areas should be removed to prevent them from overheating before the required temperature is reached in the structured areas. Moreover, an increased nickel layer thickness required a less powerful alternating electromagnetic field for the same heating effect and is thereby more efficient.

## 4. Discussion

The reported investigations revealed that oxygen plasma pretreatment of the DLP AM material PLASTCure Rigid 10500 (Prodways) and PVD coating of a thin titanium layer, acting as an adhesion promoting layer, has positive results on the adhesion of the electroplated nickel layer. Furthermore, the test specimens treated this way survived all the conducted environmental and tape-tests without failure, which improves the likelihood of the nickel layer withstanding the influence of the melt in the injection molding process. As previous studies have shown by, e.g., reproducing micro-channels smaller than 20 µm and with high aspect ratios, it is probable that the cuts made by laser ablation are not small enough to prevent melt from intruding between the nickel elements and thereby possibly damaging the heating layer upon ejection [18,28]. In any case, the geometry of the structured nickel layer will most certainly be visible as an elevated structure on the final polymer part, posing a potential source of defects for, e.g., a process chain using LDS technology, which is why thinner nickel layers could be preferable in these situations. Furthermore, although the inductive heating process is more efficient with thicker layers, a higher nickel layer thickness (e.g., 100 µm) proved to be very time consuming during the fabrication processes, and is thus not favorable for prototyping purposes.

It was also observed, that structuring the nickel layer, to form individual electrically decoupled elements, led to more homogenous heating. In fact, a structuring step is considered to be necessary to control the spatial energy intake and to prevent local overheating of the nickel layer as well as the mold. However, structuring the nickel layer also necessitates operating the inductor with a higher power in order to generate the same temperature in the nickel layer. Additionally, the excess nickel material surrounding the structured areas must be removed, as by default it has a larger area compared to the single tiles and is, therefore, more susceptible to heating by induction. This in turn will lead to overheating of these surfaces before a desired target temperature is reached in the tiles themselves.

As discussed in Section 3.4, the strength of the induced eddy currents depends, among other things, on the size of a metal element in the magnetic field. In addition, only the part of the magnetic flux perpendicular to the plane of the metal layer is relevant for the generation of eddy currents [27]. This leads to two conclusions. First, a more complex laser ablation layout with the aim of combining different sizes of nickel tiles might be used to create zones of different heating rates in the same electromagnetic field. Secondly, arranging the nickel tiles in various angles to the inductor (as is the case with a more or less complex cavity surface) will also lead to varying energy transfer rates and heating. Therefore, it will be necessary to develop and validate a simulation framework for the design of soft tooling with cavity-bound inductive heating elements as a second step.

Inductive heating of injection molds is usually performed between cycles by placing the inductor between the two mold halves. The integration of the inductor into the tooling is rare due to the inherent material properties of metal tools. Here, however, the high electromagnetic permeability of UV-curing polymers and the design freedom of the DLP technology, in combination with the presented method, can be used to integrate all elements needed for the variothermal process, i.e., the inductor and conformal cooling channels, into the soft tooling. This would constitute a soft tooling equivalent of the concept for a steel tooling based family mold with integrated variothermal features presented in [29].

## 5. Conclusions

A method for the soft tooling-friendly heating of cavities for injection molding via induction was proposed. The process chain to produce said tooling consists of additive manufacturing of the inserts, PVD coating of an adhesion agent and a seed layer, electroplating of the ferromagnetic nickel layer, and finally laser ablation. The laser ablation process is a means to increase the lifetime of the nickel layer as well as the efficiency of energy transfer. This process chain was investigated on 2D test specimens to find suitable process parameters backed by adhesion and environmental testing.

Of the alternatives investigated, a 20 nm titanium layer proved to be the best adhesion promoting layer in combination with oxygen plasma pre-treated digital light processing material PLASTCure Rigid 10500 and a 50 µm thick electroplated nickel layer. A nickel layer thickness of 50 µm as a compromise between the laser ablation duration and the inductive energy transfer efficiency is suggested, which decreases with the nickel layer thickness. The inductive heating process was examined and confirmed with a thermographic camera and the laser ablation process proved to be necessary for uniform heating of the nickel layer. It was also found to be inevitable to remove the excess nickel around the laser ablated layout to prevent overheating of these structures. The remaining nickel structures were successfully heated to 150 °C and above. To conclude, the proposed method was found to be suitable for the soft-tooling friendly heating of cavities for injection molding. Further investigations must be carried out to validate the method in an injection molding process.

## Figures and Tables

**Table 1:** Process parameters of the physical vapor deposition, oxygen plasma pretreatment and electroplating processes.

Physical Vapor Deposition Process			
Targeted thickness adhesion promoting layer (Ti, Cr)	20 nm		
Targeted thickness NiV	200 nm		
Magnetron power	500 W		
Starting pressure	5.0 × 10^−6^ mbar		
Process pressure	6.0 × 10^−4^ mbar		
Process gas/flow rate	argon/11 sccm		
**Oxygen-Plasma Pretreatment**			
Process time	360 s		
Process gas	oxygen		
Process pressure	1.5 × 10^−2^ mbar		
**Electroplating Process**			
Ni Electrolyte	LECTRO-NIC HAR-1000		
Targeted thickness Ni	15 µm	50 µm	100 µm
Process time	33 min	110 min	220 min
Electrolyte temperature	55 °C		
Current density	5 A/dm²		

**Table 2:** Results of the parameter variation of the laser ablation process. The table gives an overview of the number of passes needed to fully ablate the electroplated nickel layer in the desired area depending on layer thickness, cut width and laser power.

Ni Layer Thickness in µm	Cut Width in µm	Laser Power:	2 W	4 W	6 W	8 W
15	50	**Number** **of** **passes**	20	18	14	11
15	100		30	25	20	14
15	200		30	25	25	20
50	50		240	110	80	80
50	100		220	100	70	70
50	200		245	100	75	70
100	50		360	220	190	160
100	100		340	220	180	150
100	200		420	240	200	160
